# 

**Published:** 1904-07-02

**Authors:** 


### NOTICE TO CORRESPONDENTS.

All MSS., Letters, Books for Review, and other matters intended for the Editor should be addressed THE EDITOR, "THE HOSPITAL," THE HOSPITAL BUILDINGS, 28 & 29 SOUTHAMPTON STREET, STRAND, W.C.

The Editor cannot undertake to return rejected MS., even when accompanied by stamped directed envelope.

### Notice to Advertisers and Subscribers.

All Advertisements, Orders for Copies of THE HOSPITAL, and Business Communications should be addressed to **THE MANAGER (Not to the Editor)**, THE HOSPITAL, 28 & 29 Southampton Street, Strand, London, W.C.

The Cost of Subscription, post free, is as follows:—Per week, 3½d.; per quarter, 3s. 3d.; per half-year, 6s. 6d.; per annum, 13s. Foreign and Colonial:—Per annum, 19s. 6d., payable, in all cases, in advance.

**NOTICE.—Vol. XXXV. of "The Hospital" (October 1903 to March 1904), in handsome cloth binding, is now on sale at the office of this Journal, price 10s. 6d. Cases for binding the Numbers contained in this Volume 2s. Index to Vol. XXXV., 3½d., post free.**

**The Monthly part for July is now ready. Price 1s., by post, 1s. 3d.**

### MEDICAL APPOINTMENTS.

Edmund C. Bevers, M.A., M.B., B.Ch.Oxon., M.R.C.S., L.R.C.P., Honorary Assistant Surgeon to the Radcliffe Infirmary, Oxford. J. M. Bryan, M.R.C.S., L.R.C.P.Lond., Public Vaccinator St. Giles's and St. Andrew's Districts of the Northampton Union. Austin Cooper, B.A., M.D., B.Ch., Honorary Assistant Anæsthetist to St. John's Hospital for Diseases of the Skin, Leicester Square. W. T. Crawford, M.B., Medical Officer to the Worksop Union Infirmary. Henry J. Davis, M.A.Cantab., etc., M.R.C.P.Lond., Assistant Physician in charge of the Throat and Ear Department, West London Hospital. F. R. Eddison, M.R.C.S., L.R.C.P.Lond., Medical Officer of Health, Bedale Rural District. Robert Fielding-Ould, M.A., M.D.Oxon., Pathologist to the City of London Hospital for Diseases of the Chest. K. Fraser, M.B., Certifying Factory Surgeon for the Orrell District, Lancaster. H. Overton Hobson, M.D., Egyptian Government Inspector to the Baths and Springs, Helouan, Egypt. G. S. Leslie, M.B., C.M.Edin., Medical Officer of Health to the Failsworth Urban District. F. H. McCaughey, M.B., B.S.R.U.I., Resident Assistant Medical Officer to the Bradford Union Workhouse. J. S. McCracken, M.B., C.M.Edin., Honorary Physician to the Hospital for Sick Children, Newcastle-upon-Tyne. W. M. Montgomery, M.B., D.P.H.Camb., District Medical Officer of Health for the Eastern Transvaal. D. Moodie, L.R.C.P.&S.Edin., L.F.P.S.Glasg., District Medical Officer of the Durham Union. R. G. Nesbitt, L.R.C.P. & S.I., Medical Officer of Health to St. Just District Council. T. Richards, L.R.C.P., M.R.C.S.Eng., District Medical Officer of the Cardiff Union. George Wm. Thompson, M.B., C.M.Edin., F.R.C.S.Eng., Surgeon to the Western Ophthalmic Hospital, Marylebone Road. J. R. Hall Walker, M.D.Edin., Medical Officer and Public Vaccinator for the Fourth District of the Risbridge Union. R. M. Williams, M.B., District Medical Officer of the Bangor and Beaumaris Union. W. I. de Courcy Wheeler, Visiting Surgeon to Mercer's Hospital, Dublin. J. Hamilton Wilkes, L.D.S., R.C.S., Honorary Dental Surgeon to St. John's Hospital for Diseases of the Skin, Leicester Square.

**"The Hospital" Institutional Library.**

"Hospitals and Asylums of the World." 4 Vols., with a Portfolio of Plans, complete £12 12s.

"Burdett's Hospitals and Charities, 1904." 5s. net.

"Burdett's Official Nursing Directory." 3s. net.

"Cottage Hospitals, General, Fever, and Convalescent." 10s. 6d.

"Uniform System of Accounts for Hospitals and Public Institutions." New Edition. 4s. net.

"Hospital Expenditure: The Commissariat." 2s. 6d.

"A Legal Handbook for the Use of Hospital Authorities." 2s. 6d.

"The Cottage Hospital Case Book or Register of Patients." 10s. and 12s. 6d.

All these are published by the Scientific Press, Ltd., and may be obtained through any bookseller, or direct from the publishers 28 and 29 Southampton Street, Strand, London, W.C.

### Useful Hand-Books for Nurses.

### 1/- SERIES.

### FEVERS AND INFECTIOUS DISEASES:

The Nursing and Practical Management.

By William Harding, M.D.Ed.

### THE COMMONWEALTH OF THE BODY.

By G. A. Hawkins-Ambler, F.R.C.S.

### HOME NURSING OF SICK CHILDREN.

By J. D. E. Mortimer, M.B., F.R.C.S.

### THE CARE OF CONSUMPTIVES.

By W. H. Daw, M.R.C.S., L.R.C.P.Lond.

### NOTES ON PHARMACY AND DISPENSING FOR NURSES.

New and Revised Edition.

By C. J. S. Thompson.

### THE MIDWIVES' POCKET BOOK.

By Honnor Morten.

### THE EXAMINATION OF THE URINE.

By J. K. Watson, M.D.

**1s. 1d.** post free.

### 6d. SERIES.

### ON PREPARATION FOR OPERATION IN PRIVATE HOUSES.

By Stanmore Bishop, F.R.C.S.

### MODERN NURSING IN PRIVATE PRACTICE.

By Sir William Bennett, K.C.V.O., F.R.C.S.

**7d.** post free.

### THE SCHOTT TREATMENT FOR CHRONIC HEART DISEASES.

By Richard Greene, F.R.C.P.Ed.

### THE INSANE: THE ASYLUM AND THE NURSE.

By Richard Greene, F.R.C.P.Ed.

### ON SOME ASPECTS OF MEMORY.

By John Aikman, M.D.

### SANATORIUM TREATMENT OF CONSUMPTION.

By T. N. Kelynack, M.D., M.R.C.P.

**7d.** post free.

### "SIMPLEX" DISTRICT NURSES' CASE-BOOK.

Containing space for 50 Cases, Ruled and Printed.

Bound in Cloth Boards. **7d.** post free.

COMPLETE CATALOGUE ON APPLICATION.

*Of all Booksellers, or of*

THE SCIENTIFIC PRESS, LTD., 28 & 29 Southampton Street, Strand, LONDON, W.C.

